# Prospective cold metal working and analysis of deformation susceptibility of CuMg alloys with high magnesium content

**DOI:** 10.1038/s41598-024-57083-1

**Published:** 2024-03-18

**Authors:** Paweł Strzępek, Małgorzata Zasadzińska

**Affiliations:** Faculty of Non-Ferrous Metals, AGH University of Krakow, Al. Mickiewicza 30, 30-059 Krakow, Poland

**Keywords:** Copper alloys, CuMg, Metal working, Deformation, Hollomon, Materials for devices, Materials for energy and catalysis, Structural materials, Techniques and instrumentation, Mechanical engineering

## Abstract

Metal alloys designated for cold metal working exhibit much higher strength properties than pure materials due to solid-solution hardening. However, with the increase of mechanical properties its plasticity and workability decreases. Constant development and demand in this area has led to research on many copper alloys, such as copper alloys with high content of magnesium which were never tested before. The limitations regarding cold metal working of CuMg alloys is the main objective of this paper. Here we show that the tested materials exhibit much higher mechanical properties than currently used as electric conductors and carrying-conducting equipment materials such as pure copper, aluminum, M63 brass or CuNiSi alloy. The results were obtained using Hollomon relation, Considére criterion, Gubkin method and hardness measurements. It lead to assessing the prospective cold metal working of CuMg alloys with 2 wt% of magnesium up to 4 wt% of magnesium. The test range included upsetting with 10–50% of cold deformation. It provided the results on evolution of mechanical properties and deformability of tested alloys. Additional information was provided based on the alloys subjected to 50% of strain. The results have proven that as the amount of magnesium increased so did the assessed values, however, it was also linked with increasing friction coefficient. Measured hardness was 2 times higher and calculated Ultimate Tensile Strength (UTS) was even 2.5 times higher in reference to pure copper in the as-cast state. However, with magnesium content at 3.6 wt% or higher, the elevated amount of α + β phase causes brittleness making it impossible to subject these materials to cold metal working processes. We anticipate our assay to be a starting point for more sophisticated models and experimental research concerning cold metal working processes of CuMg alloys of high-strength, which may lead to developing novel and promising set of alloys.

## Introduction

Copper as pure material has countless applications concerning widely understood conductance of electricity or heat. However, as there are more and more demanding requirements regarding mechanical properties of certain products it turns out that pure copper is not always sufficient as its mechanical properties are limited^1,2^. These are, among others but not limited to, electromagnet windings^3,4^, medical equipment^5^, heat exchangers^6,7^ or automotive industry applications^8^.

Many alloys based on copper matrix such as CuAg alloys^9,10^, CuAl alloys^11^, CuZn alloys^12^, CuCrZr alloys^13^ or CuNiSi alloys^14,15^ are widely used nowadays in these branches of industry. Each of them is described as high strength copper alloy. The main downside regarding these alloys is high price or limited availability of most materials used to synthesize these alloys, i.e. Ag, Cr, Zr, Ni.

Many researchers have taken upon the non-conventional metal working processes such as cryogenic rolling of metals and metal alloys. This might be one of the answers to improving the mechanical properties of materials without using alloy additives that are expensive or of limited availability^16,17^. Other innovative ways of metal working studied in recent literature are for instance close die precision forging^18^, continuous extrusion process such as KOBO^19^ or accumulative angular drawing process (AAD)^20^. However, before using alternative ways of metal working it is crucial to obtain the batch materials, which is usually provided in semi-continuous or continuous casting processes. Both procedures require metallurgical synthesis in the first place.

In order to significantly lower the manufacturing cost cheaper alloys such as CuMg have been introduced. As for now only materials with maximum of 0.7 wt% of magnesium are being produced on a large industrial scale and are mainly used for overhead contact wires in railway systems and for overhead power transmission cables^21,22^. Among the researchers from around the world these materials are mainly discussed regarding the materials with low magnesium content as well (not exceeding 2 wt%)^23–25^. However, published papers mostly discuss proof strength and electrical properties of alloys subjected to heat treatment. In recent times, a few research papers have been published regarding copper alloys with higher amount of magnesium. The authors of these papers claim that with proper heat treatment it is possible to obtain Yield Strength of over 1000 MPa^26^ and that two-phase materials may maintain electrical conductivity of approximately 50 IACS % (International Annealed Copper Standard)^27^. Based on these works it can be stated that metallurgical synthesis and heat treatment of these alloys is possible and suggest that CuMg alloys may be subjected to precipitation hardening while maintaining a satisfactory level of electrical conductivity. Nevertheless, for the copper alloys with high magnesium content to be a prospective substitute material for other, more expensive alloys it is necessary to confirm their susceptibility to cold metal working, which will further increase their mechanical properties due to strain hardening. Despite copper magnesium alloys being promising materials the lack of information regarding their metal working is a huge knowledge gap which needs to be clarified, thus, making this research essential for further development of materials science considering copper alloys.

The aim of this paper is to provide information on the UTS, strength coefficient, strain exponent and friction coefficient of CuMg alloys. The results were obtained in upsetting tests which is an absolute novel approach in terms of research regarding CuMg alloys and will determine whether the materials may be subjected to metal forming processes. This type of procedure was chosen as all necessary results could be obtained from one test stand and small samples which tensile testing could not provide. What is more the materials were in the as-cast state and subjecting them to tensile testing without prior homogenization might result in false conclusions. Additionally, hardness of tested materials was evaluated to assess how much the alloys strengthen with applied strain. Obtained results were confronted with widely used materials for electrical purposes such as pure aluminum (EN AW 1050), CuZn alloy (M63 brass) and CuNiSi alloy which were obtained and subjected to analogical tests as CuMg alloys in question. What is more the employed methods were fully explained and along with the obtained results may function as a guideline for other works regarding deformability of metals and alloys.

## Experimental procedure

**Figure 1:**
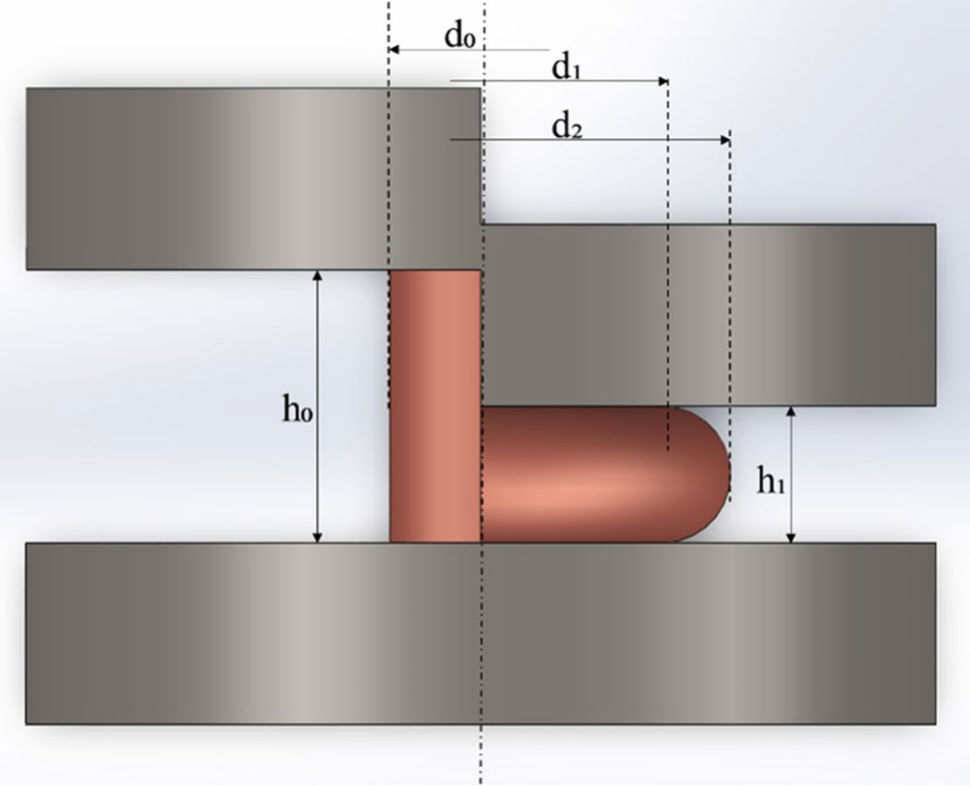
The shapes and dimensions of the cylindrical sample subjected to upsetting tests (Gubkin’s method of assesing the friction coefficient).

**Figure 2:**
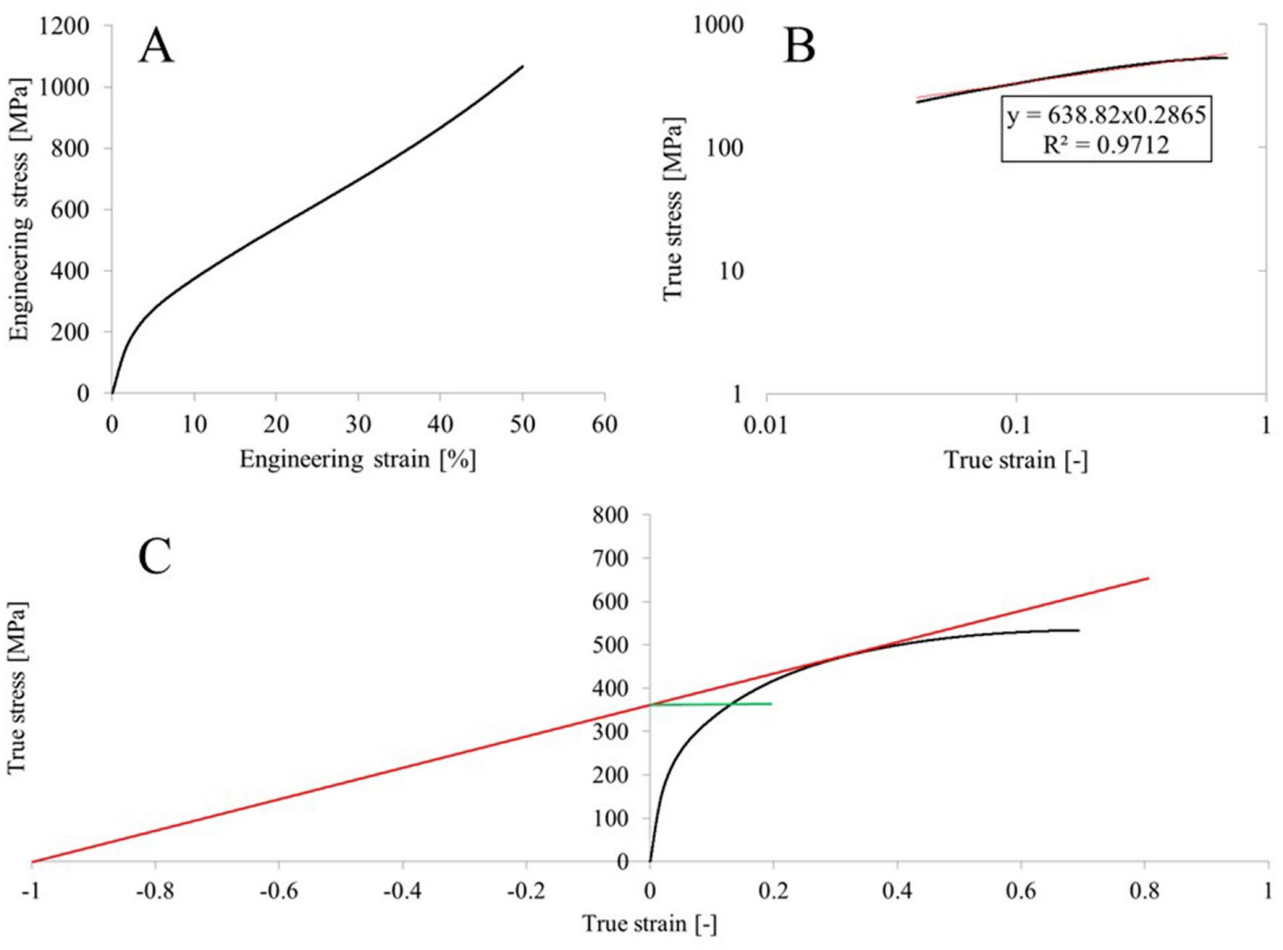
Analysis of the upsetting stress/strain relation (**A**), using Hollomon relation (**B**) and Considére criterion (**C**).

The crucial feature of metals and alloys, especially designed for conductive applications, is among others their susceptibility to metal working processes. This is mainly caused by the fact that conducting materials originate mainly from continuous or semi-continuous casting processes and further extrusion, forging or drawing. The tested copper magnesium alloys with the magnesium content ranging from 2 to 4 wt% have been obtained using laboratory horizontal continuous casting stand. The metallurgical synthesis was conducted at 1473 K with casting velocity of approximately 180 mm/min (2 s of standstill for each 6 mm of pull). The casted rod was axi-symmetrical with a diameter of 14 mm. The furnace consisted of graphite crucible and graphite crystallizer. Primary cooling system was made of copper with closed circulation of cooling medium provided at the constant velocity of 0.5 l/min. Further secondary cooling was supplied directly onto the cast rod’s surface. Additionally, the reference material of pure copper was obtained with the same casting parameters. Materials in the as-cast state were subjected to chemical composition analysis using arc spark spectrometer Spectrotest and Scanning Electron Microscopy (SEM) observations using Hitachi SU-70 microscope. The main part of the proposed research analysis consisted of subjecting the obtained materials to upsetting tests in ambient temperature with the deformation of 10%, 20%, 30%, 40% and 50%. The samples were cylindrical with diameter of 10 mm and height of 15 mm. The longer dimension was parallel to the casting direction. The upsetting tests were conducted using Zwick/Roell Z100 testing machine. The force load accuracy of the machine is 0.5% and the velocity was 1 mm/min. The tests were carried out with petroleum-based mineral oil as lubrication. The strain hardening was expressed as an average value of 5 hardness measurements obtained using Brinell’s method with Nexus 3001 tester, which test force accuracy is 0.5% and display resolution is 0.1 HB. Upsetting stress during the cold deformation process was also analyzed. Additionally, the samples subjected to 50% of strain were further analyzed in order to calculate:

- The friction coefficient employing the Gubkin’s method as presented at Fig. 1 and Eq. (1)^28^,
- UTS using Considére criterion^29,30^, which allows its assessment as the value projected at the Y axis from the tangent (red line) drawn from the negative one value to the true stress/strain upsetting curve as presented at Fig. 2C,
- Strain hardening exponent and strength coefficient as proposed by Hollomon^31^ which was collectively presented at Fig. 2 on the exemplary upsetting stress/strain relation and Eq. (2).

The microstructure of the samples subjected to 50% of strain was also analyzed in order to determine visible cracks. Electrical conductivity was measured using the eddy current device which determines the electrical conductivity of nonferrous metals expressed in MS/m. The tests were conducted on all samples after placing them for 24 h at ambient temperature in order to stabilize their thermal state which prevents the influence of temperature on measurements. 10 measurements were performed on each sample with a frequency of 60 kHz, which provides maximum depth of signal penetration. The accuracy of the equipment is 0.5% of measured values. The average values were calculated and presented as final results,

1 $$ \mu  = \left[ {\frac{{6.25 \cdot \theta  \cdot \left( {1 - \theta } \right)}}{{1 + \varepsilon }}} \right] \cdot \left( {\frac{{d_{0} }}{{h_{0} }}} \right)^{{3/2}}  $$

where µ is the friction coefficient, θ is the dimensional barreling ratio equal to (d_2_–d_1_)/d_2_, ε is the engineering strain and d_0_, d_1_, d_2_, h_0_ and h_1_ as marked at Fig. 1,

2 $${\sigma }_{s}=K\bullet {{\varepsilon }_{t}}^{n}$$

where σ_s_ is the deformation resistance, K is the strength coefficient, ε_t_ is the true strain and n is the strain hardening exponent.

## Results and discussion

The obtained results made it possible to determine the cold metal working potential of newly developed alloys which is to be presented and discussed in the following part of this work. Since magnesium boiling temperature (1364 K) is nearly identical to the melting point of copper (1357 K) it was fundamental to determine whether the obtained alloys have the assumed chemical composition as magnesium tends to evaporate easily during the metallurgical synthesis. The chemical compositions were selected to provide 3 single-phase (Cu2Mg, Cu2.4Mg and Cu2.8Mg) and 3 two-phase materials (Cu3.2Mg, Cu3.6Mg, Cu4Mg). Additional alloy with 3 wt% of magnesium (the amount where at 999 K α + β phase should occur according to^32^) and pure copper as reference material were produced. Thus, 8 materials were obtained in the continuous casting process. Each material was subjected to chemical composition analysis, which results were presented in Table 1. Providing, that the chemical composition does not vary more than 0.05 wt% (500 ppm) from the nominal values it would appear that the alloys were obtained with the amount of magnesium as intended. The amount of impurities which were present at the cast material did not exceed 50 ppm per individual element.

**Table 1:** Chemical composition analysis of the obtained alloys and estimated amount of α + β-phase at SEM images.

Nominal alloy	Cu		Cu2Mg		Cu2.4Mg	Cu2.8Mg	Cu3Mg	Cu3.2Mg	Cu3.6Mg	Cu4Mg
Cu	[wt%]		99.9	97.9	97.6	97.2	97	96.8	96.3	95.9
Mg			**0.0009**	**1.98**	**2.35**	**2.78**	**2.95**	**3.2**	**3.63**	**3.98**
Impurities			Rest							
The amount of α + β-phase	[%]		**0.000**	**6.614**	**8.267**	**10.562**	**11.637**	**15.677**	**20.319**	**26.255**

Significant values are in [bold].

**Figure 3:**
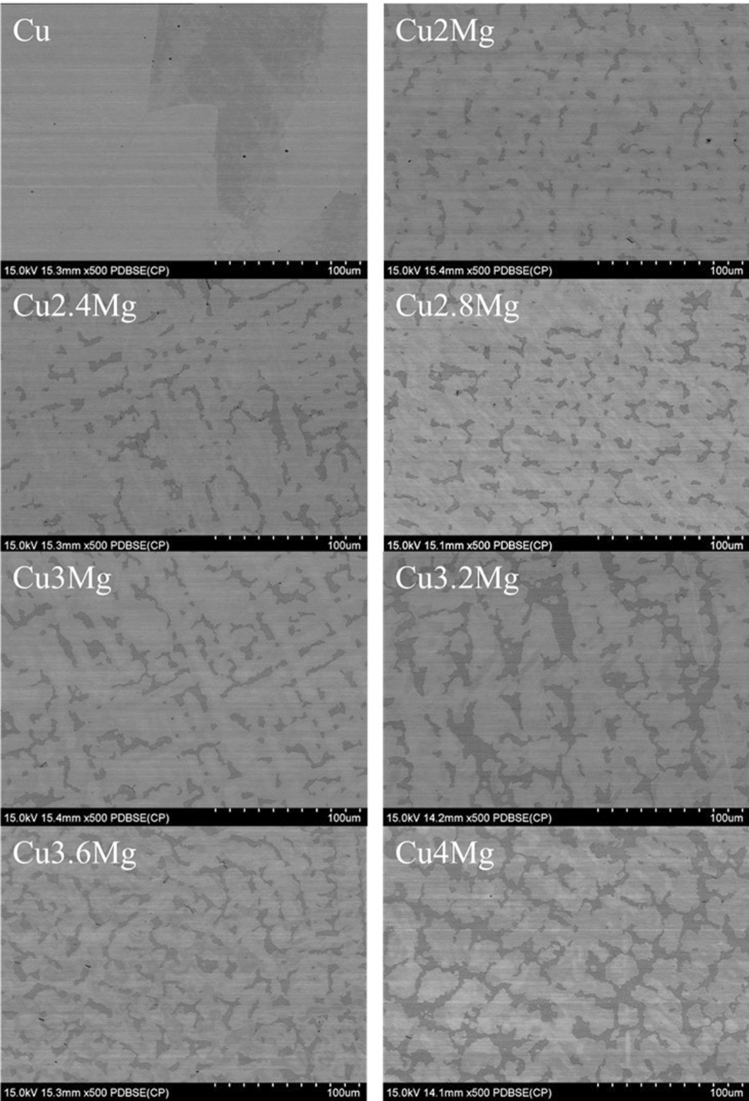
Backscatter electron SEM analysis of copper magnesium alloys; in the as-cast state; magnification ×500.

**Figure 4:**
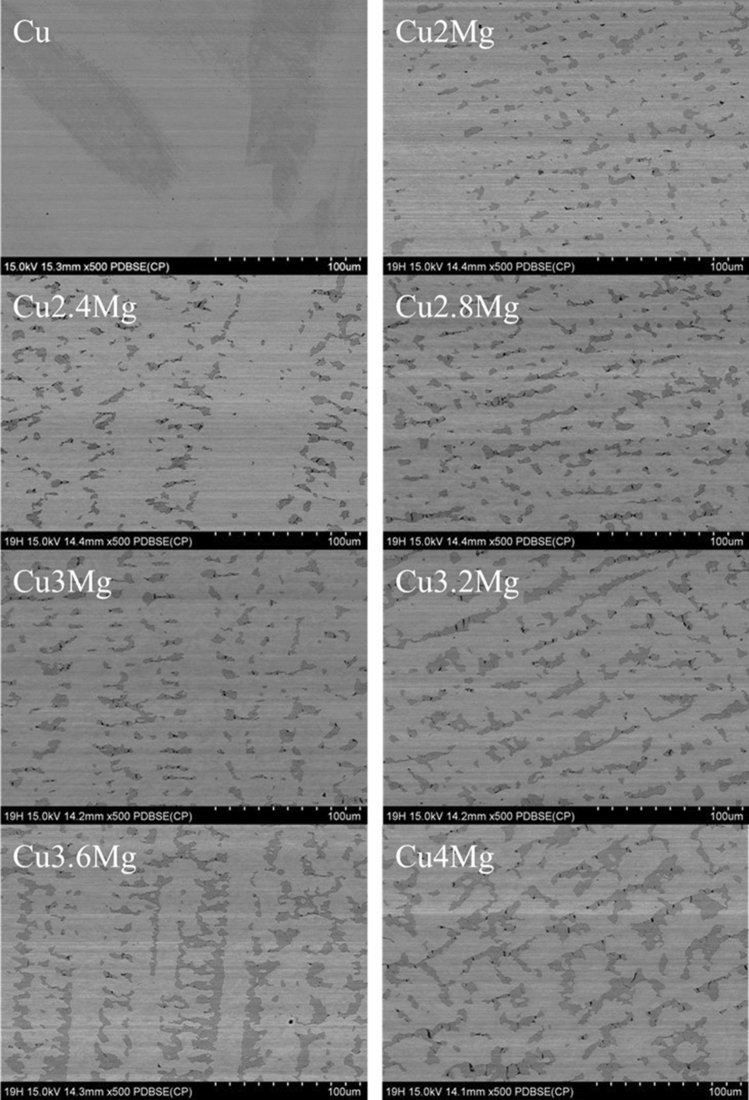
Backscatter electron SEM analysis of copper magnesium alloys; uspetting with 50% of strain; magnification ×500.

SEM observations presented in Fig. 3 prove that as the amount of magnesium increased (Table 1) the amount of α + β-phase (dark area)^27^ increased as well. It is evenly distributed among the volume of all of the materials. Using ImageJ software the amount of α + β-phase was estimated and the percentile results were placed in Table 1. The data received show that the amount of α + β-phase quadruples as the amount of magnesium increases from 2 to 4 wt%. Rapid increase starts when the amount of magnesium is higher than 3 wt%, i.e. when the alloys become two-phase materials, according to CuMg phase diagram^32^. The magnesium is not present in pure copper and it was confirmed both in chemical composition and microstructure analysis. Additional SEM observations were conducted regarding the materials subjected to 50% of strain which was presented at Fig. 4 and as presented the α + β-phase is less random and more aligned than in the as-cast state due to the applied force in a set direction.

**Figure 5:**
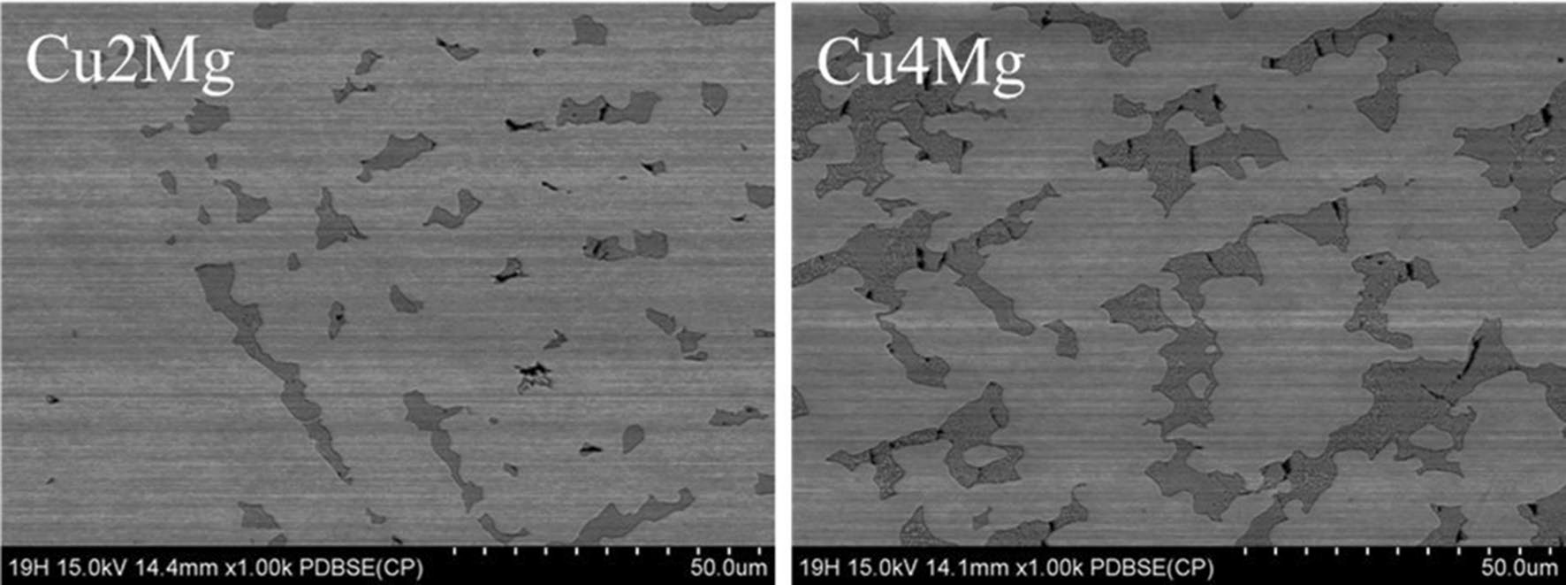
Backscatter electron SEM analysis of copper magnesium alloys; uspetting with 50% of strain; magnification ×1000.

Alloys with 2 and 4 wt% of magnesium were presented with higher magnification at Fig. 5 in order to better display the cracks resulting from compression force at a microscale. There are less than 10 cracks across Cu2Mg sample and around 40 across Cu4Mg sample. Cracks are mostly vertical, parallel to the direction of applied force and do not appear in areas where there is no α + β-phase. This would suggest that excessive amount of magnesium causes brittleness during cold metal working.

**Figure 6:**
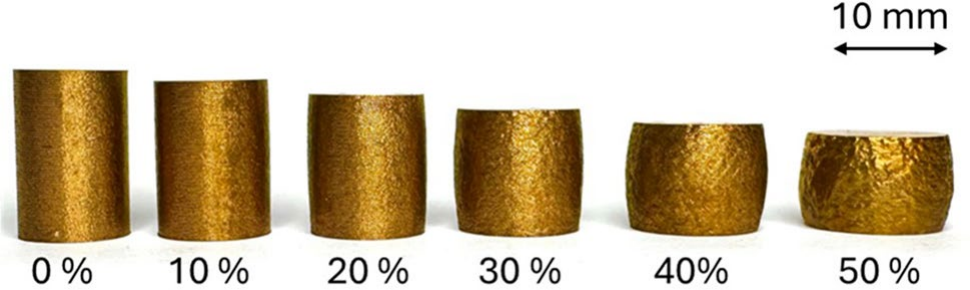
Exemplary photograph of samples subjected to upsetting tests with specified strain values.

The samples were subjected to 5 different deformation values as discussed above. An exemplary photograph of one set of samples after upsetting tests with a starting sample on the left were presented at Fig. 6 along with the strain values. The picture shows how much the samples deformed during the tests.

Since pure copper does not contain any additives it should exhibit significantly lower mechanical properties than the tested alloys. Bearing that in mind the values obtained from the upsetting tests and hardness measurements of pure copper were presented separately in Table 2. The results were presented for each of the applied upsetting strains and both values increased quasi-linearly. Since pure copper strengthens when being subjected to strain the increase in measured values of hardness was expected. This is a result of strain hardening which exhibits during metal working conducted below the temperature of recrystallization of materials^33^.

**Table 2:** Analyzed properties of pure copper.

	HB2.5/62.5						Upsetting stress [MPa]				
ε [%]	0	10	20	30	40	50	10	20	30	40	50
Cu	50.9	60.2	66.3	73.2	79.2	87.5	155	252	326	438	524

*HB* Brinell hardness, *ε* engineering strain.

**Figure 7:**
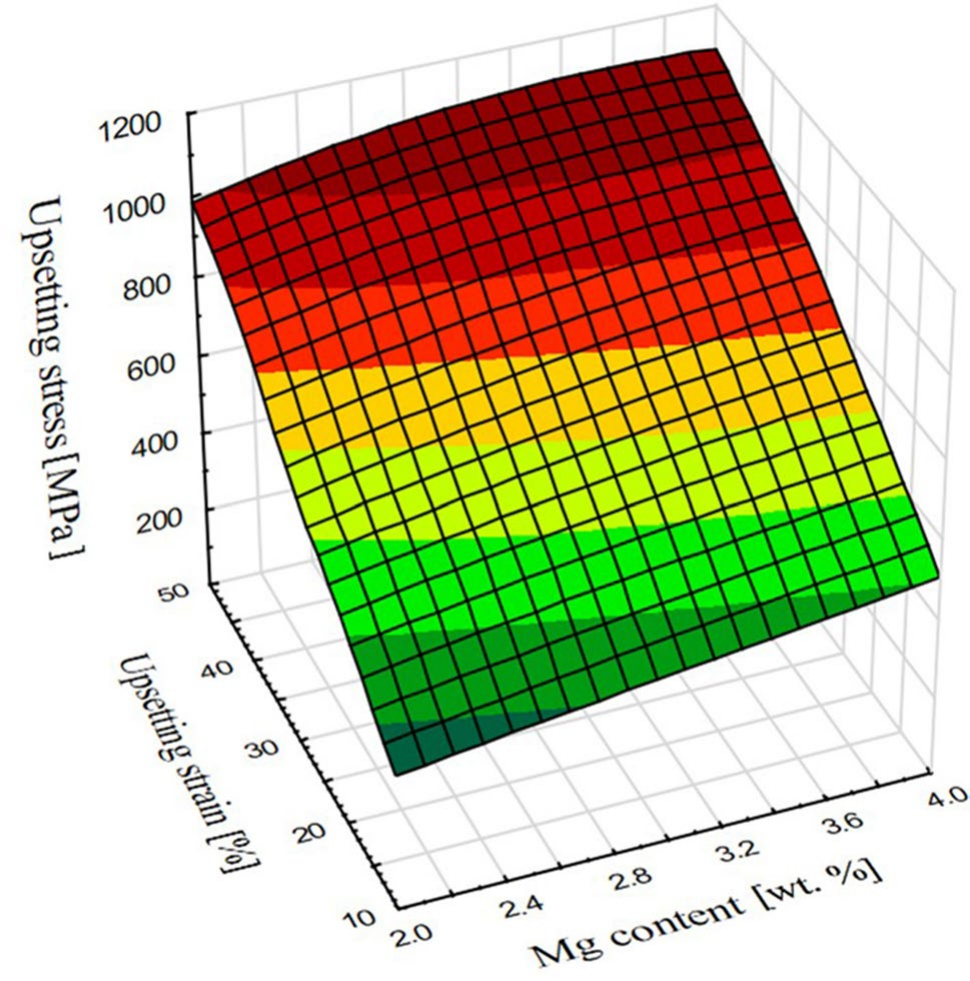
Maximum recorded upsetting stress for each of the tested alloys and applied strains.

As there was much more data regarding the tested alloys than in the case of pure copper both maximum recorded stress in upsetting tests and hardness values were presented in the form of 3-axis graphs. Maximum upsetting stress increased significantly for each material as the strain increased with the values exceeding 1000 MPa at 50% of applied cold deformation as presented at Fig. 7. The reference value of pure copper was only 524 MPa meaning that the tested alloys needed two times more force to obtain the same height reduction during compression. Remarkable was the fact that regarding single-phase materials (up to 3 wt% of magnesium) when the strain increased from 10 to 50% the recorded stress increased 3 times, whereas in the case of two-phase alloys (above 3 wt% of magnesium) it was only 2 times. However, it was notable that when the cold deformation of 50% was applied there was no difference in recorded values in the case of two-phase materials. This might mean that the amount of α + β-phase and discussed cracks is so high across the volume of the material that it could lower the stress needed to obtain the 50% height reduction. Even though, the cracks are not visible with naked eye they lower the integrity of the material.

**Figure 8:**
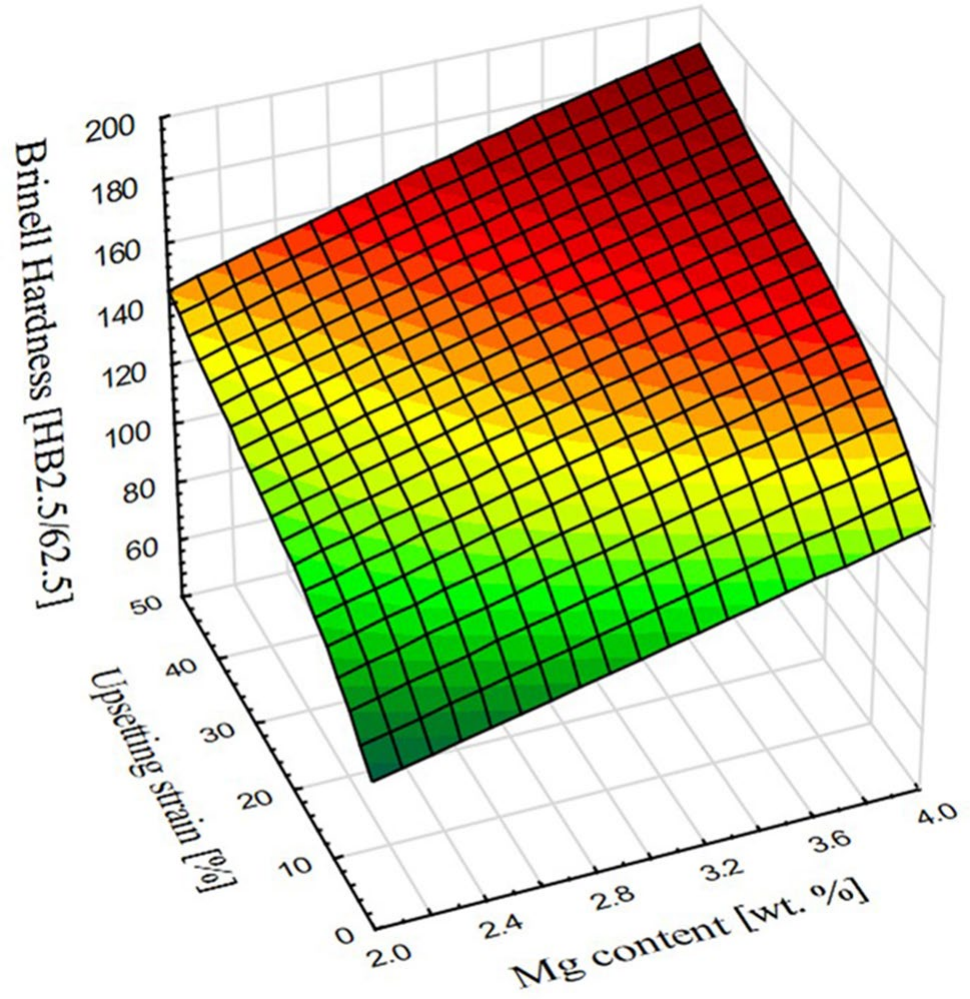
Average values of Brinell hardness for each of the tested alloys and applied strains.

The average values of hardness of samples after upsetting with given strains measured with Brinell’s method were complemented with hardness values of non-deformed material as presented at Fig. 8. The obtained results proved that there is a significant solid-solution strengthening as the hardness values of the base material increased by 80–150% (depending on the magnesium content) in reference to pure copper. The standard deviation of the obtained results was between 0.832 and 1.871 for materials in the as-cast state and up to 20% of applied strain. At higher amounts of deformation the standard deviation has risen to the range between 2.012 and 3.143. Further increase of the measured values as the deformation was applied proved that as typical copper alloys the newly developed materials may be subjected to strain hardening and the hardness increases quasi-linearly regardless of the magnesium content.

**Figure 9:**
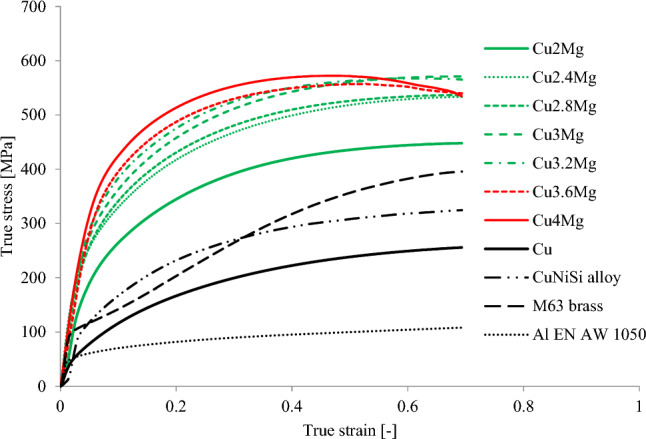
Collective upsetting true stress–true strain curves of materials subjected to 50% of strain.

Further analysis of the stress/strain relations was conducted on the compression curves obtained from the tests with 50% of applied strain, as there was the most data to analyze and the conducted calculations would reflect the actual state of material. The data obtained from the analysis as exemplary presented at Fig. 2 and collectively at Fig. 9 was presented in Table 3. For comparison, other common materials used as electric conductors and carrying-conducting equipment such as M63 brass, CuNiSi alloy and aluminum (EN AW 1050) also in the as-cast state were subjected to upsetting tests with 50% of strain. Just by analyzing the data presented at Fig. 9 it is visible that regarding Cu3.6Mg and Cu4Mg alloys (marked with the red color at the graph), when the maximum true stress is achieved its value decreases over time. Such decrease was not recorded in terms of CuMg alloys with magnesium content of up to 3.2 wt% (marked with the green color). This data correlates well with the results presented at Fig. 7 where at 50% of applied strain the maximum stress values did not increase at higher magnesium content. This may be caused as mentioned above by excess amount of α + β-phase which makes the material too brittle to sustain cold deformation of such high strain. Materials of lower magnesium content more or less remain at a constant level of true stress when reaching its maximum value. Regarding the reference materials which are marked with the black color at the graph it is visible that all materials exhibit much lower levels of true stress at 50% of applied strain than the tested CuMg alloys.

**Table 3:** Analyzed properties of CuMg alloys.

	K [MPa]	n [–]	R^2^ [–]	UTS [MPa]	µ [–]	ρ [MS/m]	% IACS [%]
Cu2Mg	542.08	0.3026	0.9604	318	0.184	26.696	46.0
Cu2.4Mg	638.82	0.2865	0.9712	367	0.206	25.154	43.4
Cu2.8Mg	641.95	0.2711	0.9584	375	0.21	24.502	42.2
Cu3Mg	684.03	0.272	0.9598	400	0.227	24.052	41.5
Cu3.2Mg	668.49	0.2336	0.9392	413	0.236	22.88	39.4
Cu3.6Mg	706.37	0.2526	0.9526	423	0.241	22.346	38.5
Cu4Mg	699.43	0.2063	0.9547	439	0.254	22.138	38.2
Cu	330.01	0.4525	0.9795	163	0.158	57.542	99.2
M63 Brass	460.15	0.4458	0.9798	241	0.232	12.368	21.3
CuNiSi	408.78	0.3883	0.9679	215	0.221	11.416	19.7
Al EN AW 1050	116.47	0.2182	0.9989	74	0.302	35.348	60.9

*K* strength coefficient, *n* strain hardening exponent, *R*^*2*^ the coefficient of determination, *UTS* ultimate tensile strength, *µ* coefficient of friction, *ρ* electrical conductivity, *IACS* International Annealed Copper Standard.

The assessed value of UTS of pure copper in the as-cast state was at the level of experimentally obtained values in^33,34^. Similarly in terms of K and n factors which regarding pure copper were close to values obtained by Bowen et al.^35^ and El-Domaity et al.^36^. Meaning that chosen methods for assessing these parameters were correct and should provide proper results for other materials. Regarding CuMg alloys the assessed UTS values using Considére criterion proved that adding just 2 wt% of magnesium to copper might increase this value almost two times, and by further increasing the magnesium content the UTS value would increase as well. The obtained results correspond very well with the hardness measurements presented at Fig. 8 and might suggest that the assumptions are correct. Regarding the values calculated using Hollomon relation (K—strength coefficient; n—strain hardening exponent) it must be noted that as the magnesium content increased the n-value decreased. It means, that formability of these alloys lowers and if given enough strain pure copper might obtain the same mechanical properties (if technologically it would be possible to do so without interoperation annealing). However, calculated K-value shows that if the applied true strain would be equal to 1 the true strength of tested alloys would be approximately 65–115% higher than this of pure copper. It proves yet again that the tested CuMg alloys with high magnesium content despite relatively lower formability would surpass pure copper in terms of mechanical properties. The calculated R^2^-value (coefficient of determination) is at relatively high level of above 0.9 (the range is between 0 and 1) meaning that the assumptions made with the trend lines predict the actual outcome quite well. However, with increased mechanical properties come disadvantages such as increased friction coefficient. That would mean that for instance in the drawing process, according to Avitzur’s analysis of the upper bound method^37^ the drawing force and thus the drawing stress would increase significantly. As copper and its alloys are mostly used as conductors of electrical energy it was necessary to measure the electrical conductivity values (ρ) and calculate the IACS %. The results presented in Table 3 show that as the amount of alloy additive increases, the electrical conductivity decreases in regard to pure copper. The standard deviation of the obtained results of electrical conductivity in MS/m was approximately 0.097 for copper, 0.083 for aluminum and between 0.035 and 0.073 for copper alloys. Regarding other commonly used copper alloys it was proven not only that they have lower electrical conductivity but also significantly lower calculated UTS, and K-value than CuMg alloys. It suggests that CuMg alloys, regardless of the magnesium content (among the tested range) are prospectively much better materials than currently used alloys. As for pure aluminum, the electrical conductivity is higher than the tested copper alloys, but its calculated mechanical properties are even 4–7 times lower than those of tested CuMg alloys. Thus, when mechanical properties may be neglected and the materials shall function only as a conductor then pure aluminum and pure copper are sufficient. However, when the final elements are carrying-conducting equipment, then CuMg alloys are superior to currently used copper alloys and pure materials.

The most common elements which require satisfactory levels of mechanical and electrical properties are among others overhead contact lines, railway contact wires, cable terminals and connectors, brackets and handles for contact wires or railway catenary systems. These are all products of wire drawing or forging processes, where the batch material may be the outcome of casting processes or casting + rolling/extrusion^38^. As of March of 2024 the market price of magnesium is around 3450 $/mt. At the same time the prices of currently used alloy additives in materials designed for the above-mentioned applications are: around 17,500 $/mt for nickel (CuNiSi alloys), around 2650 $/mt for zinc (brass) and around 28,200 $/mt for tin (bronze)^39^. This shows that only the price of zinc is noticeably lower, while nickel and tin are significantly more valuable. However, there are 2 problems with zinc as alloy additive. One is that the properties of brass regarding UTS and electrical conductivity are approximately two times lower than tested CuMg alloys as discussed above. Secondly, most brass materials contain lead in its chemical composition (some of the grades even up to 2 wt%). And as it is now generally known lead is toxic and manufacturers try to avoid it as much as possible. Last factor that needs to be discussed is the weight of the final product. The density of magnesium (1.738 g/cm^3^) is much lower than nickel (8.908 g/cm^3^), zinc (7.14 g/cm^3^) or tin (7.31 g/cm^3^). Hence, the final products will be slightly lighter, depending on the amount of magnesium used^40^.

Presented research results were obtained in laboratory conditions for the materials in the as-cast state. Since copper alloys tend to respond significantly to heat treatment one must assume that the results would vary when the homogenization, supersaturation or artificial aging would be applied.

## Conclusions

Taking all of the above into account the following conclusions were made:

1. Newly developed copper alloys with high amount of magnesium may be obtained in the continuous casting process and might be subjected to cold metal working. These factors make its prospective applications versatile as both solid-solution strengthening and strain hardening occurred when the alloys were subjected to laboratory tests in ambient temperature with no cracks visible with naked eye. However, in terms of materials with 3.6 wt% of magnesium content and higher it was noted that the amount of α + β-phase might be too high, causing brittleness and thus making it impossible to subject these materials to cold metal working processes. This may of course change if the materials would be subjected to supersaturation before applying cold deformation.
2. Maximum recorded upsetting force was 2–3 times higher than in the case of pure copper depending on the magnesium content, however, two-phase materials at 50% of applied strain did not exhibit increase in recorded force. It might be the result of brittleness coming from excessive amount of α + β-phase.
3. Regardless of the assessing method the mechanical properties of the newly developed CuMg alloys increased as the applied strain or magnesium content increased and were several times higher than those of pure copper, however, the side effect was the increase of the coefficient of friction.
4. When compared with other commonly used as electric conductors and carrying-conducting equipment materials such as M63 brass, CuNiSi alloy and aluminum (EN AW 1050) in the as-cast state it was proven that currently used copper alloys have both electrical conductivity and mechanical properties lower than those of CuMg alloys, regardless of the magnesium content. Pure aluminum despite having better electrical conductivity has 4–7 times lower mechanical properties than tested CuMg alloys.
5. The tested materials were mostly proven to withstand 50% of cold deformation in the as-cast state and as such may be used as batch material in metal working processes. Prospectively these materials may substitute currently used copper alloys such as M63 brass or CuNiSi alloy, which properties both mechanical and electrical were proven to be inferior to CuMg alloys.

## Data Availability

The datasets used and/or analysed during the current study available from the corresponding author on reasonable request.
